# Organization and resource utilization of specialized neurosurgical care for chiasmatic–sellar tumors in a metropolitan health system: a hospital-based study from Kazakhstan

**DOI:** 10.3389/frhs.2026.1807744

**Published:** 2026-04-01

**Authors:** Yerlan Ayaganov, Gani Akhanov, Zhulduz Sadykova, Berik Isatayev, Alibek Zhanisbayev, Yerdos Algazyev, Beknur Umraliyev, Sarafaddin Mansharipov, Ray Omirzak, Ermek Dyussembekov

**Affiliations:** 1 Department of Public Health and Social Sciences, Department of Internal Medicine, Kazakhstan’s Medical University “KSPH”, Almaty, Kazakhstan; 2 Department of Neurosurgery, Public Health Department of Almaty City, Municipal State Enterprise “City Hospital №7”, Almaty, Kazakhstan; 3 Department of Neurosurgery, Kazakh National Medical University Named After S.D. Asfendiyarov, Almaty, Kazakhstan

**Keywords:** centralized care, chiasmatic-sellar tumors, health services, hospital resource utilization, Kazakhstan, neurosurgery care

## Abstract

**Background/objectives:**

Chiasmatic–sellar region tumors represent a significant burden for specialized healthcare services due to the need for complex diagnostics, multidisciplinary management, and prolonged inpatient treatment. In the absence of population-based registries, hospital-based analyses provide important insights into the organization of care and resource utilization. This study aimed to assess the organization and key characteristics of specialized neurosurgical care for patients with chiasmatic–sellar tumors in a large metropolitan center in Kazakhstan.

**Methods:**

A retrospective hospital-based study was conducted using medical records of adult patients treated for chiasmatic–sellar region tumors in a tertiary neurosurgical center in Almaty between 2019 and 2024. Demographic characteristics, tumor structure, surgical activity dynamics, length of hospital stay, intensive care unit (ICU) utilization, perioperative complications, reoperations, and in-hospital mortality were analyzed using descriptive statistical methods.

**Results:**

A total of 342 patients were included (mean age 49.6 ± 14.2 years; 59.9% women). Pituitary adenomas accounted for 82.2% of cases. Surgical activity varied over time, with a temporary decline followed by a compensatory increase in subsequent years. Median length of hospital stay was 16 days (IQR 14–20), and routine short-term ICU monitoring was required for the majority of patients. Postoperative complications occurred in 5.8% of cases, reoperations in 4.1%, and in-hospital mortality was 2.6%.

**Conclusions:**

This hospital-based study highlights key organizational features and resource utilization patterns of specialized neurosurgical care for chiasmatic–sellar tumors in a metropolitan setting. These findings provide real-world evidence to support planning and optimization of centralized neurosurgical services in metropolitan health systems of middle-income countries, particularly with regard to inpatient capacity, intensive care utilization, and standardized postoperative pathways.

## 1 Introduction

Chiasmatic–sellar region (CSR) tumors comprise a heterogeneous group of intracranial neoplasms arising in the sella turcica and optic chiasm region, including pituitary adenomas, craniopharyngiomas, skull base meningiomas, chordomas, and optic tract gliomas (1). From a public health perspective, these tumors represent a clinically and organizationally significant disease group due to their relatively high prevalence, chronic course, and requirement for specialized multidisciplinary care.

Pituitary adenomas constitute the most common type of CSR tumors, accounting for 6.5%–18% of all intracranial neoplasms (2). Population-based studies report an incidence of 3.9–7.4 cases per 100,000 population per year and a prevalence ranging from 76 to 116 cases per 100,000 (3), indicating a substantial cumulative burden on healthcare systems. A predominance of female patients has been consistently observed, which is largely attributed to the higher frequency of prolactin-secreting and clinically detectable microadenomas in women (4). Although craniopharyngiomas are rare, with reported incidence rates of 0.13–2 cases per 100,000 population per year and a standardized incidence of 0.19 per 100,000 (95% CI: 0.16–0.21) in large registries (5, 6), their management is associated with high resource consumption due to complex surgical treatment and long-term follow-up. Meningiomas occur with an incidence of 6–0 cases per 100,000 population; however, only a small proportion are localized within the chiasmatic-sellar region (7).

The public health relevance of CSR tumors is determined not only by their epidemiological characteristics but also by their clinical consequences and organizational impact on healthcare services. Compression of the optic pathways and disruption of hypothalamic–pituitary regulation frequently lead to visual impairment and endocrine dysfunction, including acromegaly, hypercorticism, hyperprolactinemia, and hypopituitarism (8–10). These conditions often result in reduced quality of life, long-term disability, and increased demand for repeated medical consultations, diagnostic procedures, and inpatient care.

Diagnosis and management of CSR tumors require advanced diagnostic technologies and coordinated multidisciplinary care. Magnetic resonance imaging (MRI) remains the standard diagnostic modality for visualizing the sellar region (11), frequently supplemented by computed tomography for assessment of bone structures and calcifications. Comprehensive hormonal evaluation and ophthalmological examination are mandatory components of patient assessment (12, 13), underscoring the need for coordinated interaction between neurosurgeons, endocrinologists, ophthalmologists, and radiologists. Over recent decades, the widespread availability of MRI and increased clinical awareness have led to higher detection rates of pituitary adenomas (14, 15), further increasing the demand for specialized healthcare services. Clinical management of sellar region tumors is often complicated by their proximity to critical neurovascular and endocrine structures. Large tertiary-center studies report that suprasellar pituitary adenomas frequently present with visual disturbances due to optic chiasm compression and that macroadenomas constitute nearly 70% of cases at diagnosis (16). In addition, rare hormonally active adenomas may demonstrate co-secretion of multiple pituitary hormones, requiring comprehensive endocrine evaluation and multidisciplinary management. In a tertiary-center series of TSH/GH co-secreting adenomas, invasive tumor growth involving surrounding structures was observed in more than 60% of cases, further highlighting the complexity of diagnosis and treatment planning (17).

Despite the growing clinical and organizational relevance of CSR tumors, national data from Central Asian countries remain limited. Published retrospective observations from Kazakhstan indicate that approximately 83% of CSR tumors are pituitary adenomas (18); however, systematic analyses describing tumor structure, patient characteristics, organization of care, and healthcare resource utilization are lacking. This gap persists despite the introduction of advanced treatment technologies, including endoscopic transsphenoidal surgery since 2008, three-dimensional endoscopy since 2017, and Gamma Knife radiosurgery since 2021 (19, 20). To date, no comprehensive assessments of surgical outcomes, complication rates, or intensive care utilization for CSR tumors have been published in Kazakhstan.

In the absence of a national population-based registry, hospital-based analyses remain a critical source of information for evaluating patterns of care delivery, inpatient resource utilization, and organizational aspects of specialized neurosurgical services. Although such studies do not allow estimation of true population incidence or prevalence, they provide valuable insights into real-world healthcare system performance, particularly in settings with centralized tertiary care and limited epidemiological surveillance.

Therefore, the aim of this study was to conduct a hospital-based assessment of clinical and demographic characteristics, tumor structure, and key organizational parameters of neurosurgical care for patients with chiasmatic–sellar region tumors treated in Almaty, the largest metropolitan center in Kazakhstan, between 2019 and 2024.

## 2 Materials and methods

### 2.1 Study design and setting

A single-center retrospective descriptive study was conducted to assess the organization, structure, and key characteristics of specialized neurosurgical care for patients with chiasmatic–sellar region (CSR) tumors. The study was performed at a large multidisciplinary tertiary hospital in Almaty, Kazakhstan, which serves as a referral center for specialized neurosurgical care. The hospital is a large tertiary multidisciplinary institution with approximately 750 inpatient beds and a workforce of more than 2,700 employees, including over 560 physicians and more than 1,300 nursing staff. The hospital structure includes specialized surgical departments such as neurosurgery, neurology, and neuro-intensive care units. According to institutional reports, more than 4,600–5,100 surgical procedures are performed annually across surgical departments, reflecting a high clinical workload and the need for coordinated work of surgical teams, anesthesiology services, and intensive care personnel.

The analysis covered the period from January 2019 to December 2024 and was based on hospital registry data from the neurosurgical department. A hospital-based design was chosen due to the absence of a national population-based registry for CSR tumors, allowing evaluation of real-world patterns of care delivery and inpatient resource utilization within a centralized healthcare setting.

Patients were referred to the neurosurgical department through several pathways typical of centralized tertiary care systems. Most patients were referred by neurologists, endocrinologists, ophthalmologists, or general practitioners from city outpatient clinics after pathology in the sella turcica was identified during diagnostic imaging and hormonal testing. In some cases, patients were admitted to the hospital urgently due to acute visual impairment or neurological symptoms. The hospital functions as a regional tertiary care center for complex neurosurgical conditions, integrating specialized expertise, advanced diagnostic technologies, and a multidisciplinary approach to decision-making.

### 2.2 Data sources and data collection

Data were obtained from multiple institutional sources, including electronic medical records, discharge summaries, surgical logs, and the hospital registry of neurosurgical interventions coded according to the International Classification of Diseases, 10th Revision (ICD-10: D35.2, D44.3, C75.1, Q04.8). Additional information was retrieved from laboratory and instrumental archives, including hormonal panels, magnetic resonance imaging (MRI), computed tomography (CT), and ophthalmological examination reports.

Data extraction was performed manually using the hospital's electronic database and standardized data collection forms. The following variables were collected: sociodemographic characteristics (age, sex, place of residence); tumor-related data (tumor type and localization); care delivery parameters (type of surgical approach, duration of surgery, perioperative complications); indicators of hospital resource utilization [length of hospital stay, intensive care unit (ICU) admission and duration]; and short-term outcomes, including reoperations and in-hospital mortality.

To ensure data accuracy and completeness, all records underwent double verification through independent review by two researchers, followed by consolidation into a single analytical dataset. In cases of discrepancy, surgical protocols and histopathological reports were considered the primary reference sources.

### 2.3 Inclusion and exclusion criteria

The study included adult patients (≥18 years) with a confirmed diagnosis of a chiasmatic–sellar region tumor based on clinical evaluation, neuroimaging (MRI and/or CT), and histopathological verification, who were hospitalized and treated surgically during the study period.

Inclusion criteria were: (1) tumor localization within the sella turcica, suprasellar, or parasellar region according to imaging data; (2) availability of histopathological confirmation; (3) completeness of medical documentation, including diagnostic, surgical, and outcome data; and (4) primary hospitalization during the study period. Patients with repeated hospitalizations were counted once, based on the initial surgical episode.

Exclusion criteria comprised non-neoplastic lesions (e.g., inflammatory, granulomatous, vascular, or aneurysmal processes), absence of histological or imaging confirmation, incomplete or inconsistent medical records, pediatric cases (<18 years), and patients with severe comorbid conditions that could substantially confound outcome assessment. Pediatric patients were excluded due to fundamentally different clinical pathways and treatment protocols. The final analytical sample included 342 patients treated between 2019 and 2024.

### 2.4 Definitions and outcome measures

In routine clinical practice, postoperative follow-up includes clinical evaluation and magnetic resonance imaging (MRI) during scheduled outpatient visits, typically within the first 3–6 months after surgery and subsequently depending on tumor type, endocrine status, and clinical condition. Due to the retrospective design of the present study and variability in follow-up duration across patients, recurrence data were interpreted descriptively rather than through formal survival analysis.

From a health services perspective, key outcome measures included indicators of care organization and hospital resource utilization, such as length of hospital stay, ICU admission and duration, reoperations within 30 days, postoperative complications, and in-hospital mortality.

### 2.5 Ethical considerations

The study was conducted in accordance with the ethical principles of the Declaration of Helsinki (2013 revision) and national regulatory requirements of the Republic of Kazakhstan governing biomedical research involving human subjects (21). The study protocol was approved by the Local Ethics Committee of the Kazakhstan Medical University of the Higher School of Public Health (KMU KSPH) (Protocol No. 9, dated 27 May 2024).

All patient data were anonymized prior to analysis. Personal identifiers, including names, medical record numbers, and dates of birth, were replaced with unique codes to ensure confidentiality. Given the retrospective and non-interventional nature of the study, individual informed consent was waived. All data were analyzed in aggregate form, and the authors assume responsibility for compliance with ethical and legal standards.

### 2.6 Statistical analysis

Statistical analysis was performed using IBM SPSS Statistics version 26.0 and Microsoft Excel 2021. All variables were assessed for completeness, logical consistency, and duplication prior to analysis.

Descriptive statistics were used to summarize the data. Quantitative variables were presented as mean (M) with standard deviation (SD) or median (Me) with interquartile range (IQR), depending on data distribution. Qualitative variables were expressed as absolute and relative frequencies (*n*, %) with 95% confidence intervals (95% CI). Normality of distribution was assessed using the Shapiro–Wilk test.

Comparisons between groups were performed using the Student's t-test or Mann–Whitney *U*-test, as appropriate. Categorical variables were compared using the Pearson *χ*^2^ test or Fisher's exact test when expected cell counts were <5. Correlations were assessed using Pearson or Spearman coefficients, depending on variable type. Statistical significance was set at *p* < 0.05. Although exploratory analyses of recurrence-related factors were performed, all inferential analyses were interpreted cautiously due to the retrospective design and variable follow-up duration, which was calculated from the date of primary surgery to the last available clinical assessment.

## 3 Results

### 3.1 Characteristics of the service population

During the study period (2019–2024), a total of 342 adult patients with chiasmatic–sellar region tumors were treated surgically in the neurosurgical department of a tertiary multidisciplinary hospital in Almaty.

The mean age of patients was 49.6 ± 14.2 years, with a median age of 51 years (IQR 38–61; range 18–77). Women accounted for 59.9% (*n* = 205) of cases, while men represented 40.1% (*n* = 137), resulting in a female-to-male ratio of 1.5:1 (Table 1). No statistically significant differences in mean age were observed between men and women (51.1 ± 14.3 vs. 48.6 ± 14.0 years; *p* = 0.117).

**Table 1:** Demographic indicators.

Indicator	Meaning
Number of patients	342
Mean age, M ± SD	49.6 ± 14.2 years
Median age (IQR)	51 (38–61) years old
Age range	18–77 years old
Women, *n* (%)	205 (59.9%)
Men, *n* (%)	137 (40.1%)
F:M ratio	1.5:1

**Figure 1:**
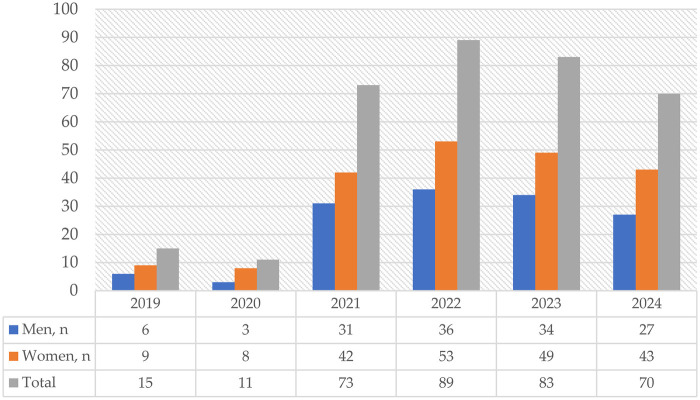
Annual volume of specialized neurosurgical care for chiasmatic–sellar region tumors by sex, 2019–2024.

The annual volume of specialized neurosurgical procedures increased substantially over time, from 15 cases in 2019 to a peak of 89 cases in 2022, followed by stabilization at 70–83 procedures per year. Across all study years, the predominance of female patients (57%–60%) was maintained, while the mean age of patients increased gradually from 45.9 years in 2019 to 50.6 years in 2024 (Figure 1).

### 3.2 Tumor structure as an indicator of case mix

Pituitary adenomas constituted the majority of cases (*n* = 281; 82.2%), followed by meningiomas (*n* = 23; 6.7%) and other benign or cystic lesions of the chiasmatic–sellar region (*n* = 7; 2.0%). Rare tumor types included chordoma (*n* = 1; 0.3%) and craniopharyngioma (*n* = 1; 0.3%). An additional 29 cases (8.5%) were classified as other lesions, including metastatic tumors (*n* = 12), mucoceles (*n* = 7), cholesteatomas (*n* = 4), and rare cystic formations (*n* = 6).

Overall tumor recurrence during the observation period was documented in 13 patients (3.8%; 95% CI: 2.2–6.4). Recurrence was least frequent among pituitary adenomas (2.5%), while higher proportions were observed among cystic and other benign lesions. Due to the small number of cases in several diagnostic subgroups, recurrence data were interpreted descriptively.

### 3.3 Surgical activity and care delivery characteristics

All surgical interventions were performed using a transsphenoidal approach (endoscopic or microscopic), reflecting a standardized institutional surgical strategy.

The mean duration of surgery was 106.1 ± 38.2 min, with a median of 100 min (IQR 80–124). Mean intraoperative blood loss was 210.6 ± 174.3 mL, with a median of 200 mL (IQR 50–300). A moderate positive correlation was observed between operative time and blood loss (*ρ* = 0.41; *p* < 0.001), indicating increased technical complexity in longer procedures.

### 3.4 Hospital resource utilization and short-term outcomes

Indicators of hospital resource utilization are summarized in Table 2. Intraoperative complications were recorded in 36 patients (10.5%; 95% CI: 7.7–14.2). Postoperative complications within 30 days occurred in 20 patients (5.8%; 95% CI: 3.8–8.9).

**Table 2:** Hospital resource utilization and short-term outcomes in patients with chiasmatic–sellar region tumors.

Indicator	Value	95% CI
Intraoperative complications, *n* (%)	36 (10.5%)	7.7–14.2
Postoperative complications ≤30 days, *n* (%)	20 (5.8%)	3.8–8.9
Reoperations ≤30 days, *n* (%)	14 (4.1%)	2.5–6.8
ICU admission, *n* (%)	339 (99.1%)	97.4–99.7
ICU stay, days, mean ± SD	1.11 ± 1.08	–
Length of hospital stay, days, mean ± SD	16.6 ± 5.2	–
Length of hospital stay, days, median (IQR)	16 (14–20)	–
In-hospital mortality, *n* (%)	9 (2.6%)	1.4–4.9

Reoperations within 30 days were required in 14 patients (4.1%; 95% CI: 2.5–6.8), most commonly due to cerebrospinal fluid leakage, postoperative bleeding, hydrocephalus, or residual tumor tissue.

Almost all patients (*n* = 339; 99.1%) required postoperative admission to the intensive care unit (ICU), reflecting routine institutional monitoring protocols rather than critical illness. The mean ICU stay was 1.11 ± 1.08 days. The mean length of hospital stay was 16.6 ± 5.2 days, with a median of 16 days (IQR 14–20).

In-hospital mortality was recorded in 9 patients (2.6%; 95% CI: 1.4–4.9). The primary causes of death included cerebral edema, severe infectious complications, thromboembolic events, and cardiovascular complications, often in combination.

### 3.5 Exploratory analysis of organizational indicators

No statistically significant differences were observed in operative duration or length of hospital stay between patients with and without postoperative complications (*p* > 0.05). Age and sex were not significantly associated with recurrence or reoperation rates.

The only consistent statistically significant association identified was between operative duration and intraoperative blood loss (*ρ* = 0.41; *p* < 0.001), reflecting procedure complexity rather than organizational inefficiency. Overall, the absence of significant associations between patient characteristics and key organizational indicators supports the stability and predictability of inpatient care delivery within the studied system.

## 4 Discussion

This hospital-based study provides a comprehensive overview of the organization and resource utilization of specialized neurosurgical care for patients with chiasmatic-sellar region tumors in a large metropolitan center in Kazakhstan. The public health relevance of such tumors is well recognized, given their relatively high prevalence among intracranial neoplasms, chronic disease course, and long-term demand for specialized care (22, 23). In the absence of population-based registries, hospital-based analyses represent a practical and informative approach for evaluating real-world service delivery and health system performance.

One of the key findings of this study is the substantial increase in the volume of specialized neurosurgical care over time, followed by stabilization in recent years. Similar trends in neurosurgical activity have been reported internationally and are often interpreted as indicators of growing diagnostic capacity, improved referral pathways, and increasing demand for specialized services (23). Temporary fluctuations in surgical volume observed during the study period are also consistent with global disruptions in elective neurosurgical care reported during the COVID-19 pandemic (24, 25), underscoring the vulnerability of centralized high-complexity services to system-level stressors.

The predominance of female patients observed in this cohort aligns with previous reports describing gender differences in pituitary adenomas, particularly related to the higher detection rates of hormonally active and microadenomas in women (26). From a health services perspective, these demographic characteristics define the population that places sustained demand on specialized neurosurgical and endocrine services, rather than reflecting true population-level epidemiology.

Hospital resource utilization represents a central dimension of care organization in this study. Nearly universal short-term postoperative admission to the intensive care unit (ICU) reflects standardized institutional protocols rather than clinical severity, a practice that has been widely described in centers performing transsphenoidal and endoscopic endonasal surgery (27). The relatively short ICU stay and stable length of hospitalization observed in this cohort suggest a predictable and standardized inpatient care pathway, which is essential for effective capacity planning and efficient use of limited critical care resources.

The rates of postoperative complications, reoperations, and in-hospital mortality were low and comparable to those reported in large institutional series (28). Importantly, these outcomes were not associated with prolonged hospitalization or increased resource utilization at the system level, suggesting that increasing service volume did not compromise short-term organizational performance. This finding supports the potential efficiency of centralized care models for complex neurosurgical conditions, as previously suggested in studies of pituitary adenomas and other sellar region tumors (23, 27).

Although rare tumor entities such as craniopharyngiomas and atypical sellar lesions accounted for a small proportion of cases in this study, their management is known to be resource-intensive and associated with prolonged follow-up and multidisciplinary care requirements (29, 30). Delays in diagnosis and referral for sellar tumors have also been identified as important contributors to disease burden and healthcare utilization, further emphasizing the need for coordinated diagnostic and referral pathways (31).

The organization of care in the studied center is based on a multidisciplinary approach involving neurosurgeons, endocrinologists, ophthalmologists, radiologists, and anesthesiologists. Multidisciplinary team-based management is widely regarded as the standard of care for chiasmatic–sellar region tumors and has been associated with improved coordination, reduced variability in care, and optimized use of hospital resources (27, 32, 33). Although the present study did not directly evaluate the impact of multidisciplinary decision-making on outcomes, the stability of key organizational indicators likely reflects the contribution of coordinated care pathways within a centralized setting.

Human resource allocation represents an important dimension of specialized neurosurgical care (34). The high surgical workload and the presence of specialized neurosurgical and neuro-intensive care units require coordinated work of neurosurgeons, anesthesiologists, operating room nurses, and ICU personnel (35). However, detailed indicators of staffing ratios and operative team workload were not systematically captured in the hospital registry used for this study.

Overall, this study demonstrates that hospital-based registry data can provide valuable insights into the organization, capacity, and performance of specialized neurosurgical services in settings with centralized care and limited epidemiological surveillance. Incorporation of standardized indicators of service volume and resource utilization into routine monitoring frameworks may support evidence-based health system planning and contribute to the sustainable delivery of high-complexity medical care in Kazakhstan and comparable middle-income countries. From a health system perspective, the observed stability of length of stay, short ICU duration, and low complication rates despite increasing surgical volume provides practical evidence to inform planning and optimization of centralized neurosurgical services in metropolitan health systems.

### 4.1 Limitations

Several limitations of this study should be acknowledged. First, the retrospective single-center design limits the generalizability of the findings to other settings and healthcare systems. The study reflects the experience of a large metropolitan tertiary referral center, and organizational patterns may differ in regional or non-specialized hospitals.

Second, the absence of a national population-based registry for chiasmatic–sellar region tumors precludes estimation of incidence and prevalence and limits the ability to assess access to care at the population level. As a hospital-based analysis, the study captures only patients who reached specialized neurosurgical services and does not account for individuals managed conservatively or referred elsewhere.

Third, follow-up duration varied among patients due to routine clinical practice and the retrospective nature of data collection, restricting the assessment of long-term outcomes and recurrence dynamics. In addition, rare tumor subtypes were represented by very small numbers, which limited subgroup analyses and necessitated descriptive interpretation of certain findings.

Finally, although organizational indicators and short-term outcomes were analyzed in detail, the study did not directly evaluate patient-reported outcomes, quality of life, or economic aspects of care, which are important components of comprehensive health system assessment. Future multicenter studies incorporating standardized registries and economic evaluations would provide a more complete picture of the organization and efficiency of specialized neurosurgical care.

## 5 Conclusions

This hospital-based study provides insight into the organization and resource utilization of specialized neurosurgical care for patients with chiasmatic–sellar region tumors in a large metropolitan center in Kazakhstan. The findings demonstrate a sustained increase followed by stabilization of service volume, accompanied by predictable patterns of inpatient resource use, including routine short-term intensive care monitoring and standardized lengths of hospital stay.

From a public health perspective, these results indicate that centralized models of care can support the delivery of high-complexity neurosurgical services while maintaining stable short-term organizational performance. In settings with limited population-based surveillance, hospital-based registry analyses represent a practical and informative approach to monitoring health system performance.

Incorporation of standardized indicators of service volume and inpatient resource utilization into routine health system planning may facilitate capacity management, improve access to specialized neurosurgical care, and support the long-term sustainability of tertiary services in Kazakhstan and comparable middle-income countries.

## Data Availability

The raw data supporting the conclusions of this article will be made available by the authors, without undue reservation.
